# Editorial: Visceral surgery and education

**DOI:** 10.3389/fsurg.2024.1548598

**Published:** 2025-01-09

**Authors:** Gabriel Sandblom, Magnus Kjellman, Lars Enochsson

**Affiliations:** ^1^ Department of Clinical Science and Education Södersjukhuset, Department of Surgery, Karolinska Institutet, Södersjukhuset, Stockholm, Sweden; ^2^ Department of Breast, Endocrine Tumors, and Sarcoma, Karolinska University Hospital, Stockholm, Sweden; ^3^ Department of Molecular Medicine and Surgery, Karolinska Institutet, Stockholm, Sweden; ^4^ Division of Orthopedics and Biotechnology, Department of Clinical Science, Intervention and Technology, Karolinska Institutet, Stockholm, Sweden

**Keywords:** education, skills training, residency, apprentice, feedback, trainee

**Editorial on the Research Topic**
Visceral surgery and education

“*Tell me and I forget, teach me and I may remember, involve me and I learn.”*—Benjamin Franklin

In many ways, the teaching of surgical skills resembles the way handicrafts have been taught for hundreds of years. In former days, the three classic levels in learning a craft were apprentice, then journeyman, and finally master. The apprentice lacked experience but gained experience by working with a master. Eventually, the apprentice gained sufficient skill to become a journeyman which meant he was able to work independently—usually jobs taking a day or so. Ideally, the journeyman wandered from master to master until he finally obtained sufficient experience and skills to become a master himself. In surgery, however, the apprentice does not work with stone, metal or wood, the young surgeon works with the tissues of human beings. There is thus a careful trade-off between the surgeon's freedom to train his/her skills and the restriction of not harming the patient.

A constant obstacle to junior surgeons gaining the skills required to go forward is the lack of training opportunities during the first years of residency. The teaching of clinical skills is seldom standardised, which leads to a wide variation in the resident's competence to perform procedures that is not necessarily due to degree of diligence. To overcome the disparity in training opportunities, the Essential Surgical Skills Course (ESSC) and Bridging the Gap Course (BtG) were introduced in the United Kingdom in 2015 Kwan et al.. The rationale behind these courses was to expose surgical trainees to core surgical training, providing basic skills that have universal application in further training. These courses include hands-on practice stations on synthetic material or animal tissues. Feedback from course participants has been generally favourable. Animal tissues are preferred to synthetic materials. Furthermore, video demonstrations were generally not so popular. Demonstrations in a low-pressure setting without time restraint, and with teachers who agree on a standardised approach to avoid contradiction has been shown to provide the highest degree of skill retention.

In healthcare in general and surgery in particular, one of the greatest preventable causes of adverse events is miscommunication between individuals and units. In a UK teaching hospital, it was shown that the handing over of patients at the end of the weekend was markedly improved after educating registrars and introducing a standardised proforma Dexter et al.. The registrar education programme required little in terms of resources but resulted in a substantial improvement in continuity of care and patient safety.

The European Association of Endoscopic Surgery encourages and supports continuous education programmes, supportive endoscopy training, and quality assurance to improve patient outcome in endoscopic surgery. These goals require prioritisation and careful planning of resource allocation. If not critically evaluated, research projects and further education may be a waste of money and even counterproductive. To fully understand the impact of funding provided by the EAES, the EAES research committee has embarked on a project to assess the extent to which the EAES research funding scheme has achieved the abovementioned goals McClean et al.. This project will be conducted using a mixed methods approach, beginning with semi-structured interviews with several previous grant recipients. Based on a thematic analysis of these interviews, a questionnaire will be designed and sent to a broader group of previous grant recipients. If the survey is to provide meaningful data, it must ideally be applicable to all situations in which a clinician seeks funding from an organisation that finances research and education.

Organising surgical training in a safe and effective way is complex. In this context, the development of virtual simulator training has added yet another tool that has simplified training, trainee performance assessment, and monitoring progress in the simulated environment. However, extrapolation of skills acquired on a virtual simulator to the real-world situation is fraught with obstacles. Safe surgery also requires communicative skills and awareness of the impact of stress on one's ability to make adequate decisions.

These challenges must be faced and the experience gained condensed into global evaluation of surgical training and skills practised in the operating theatre, as well as in decision-making and day-to-day surgical care.

